# Rectal Foreign Body Removal in the Emergency Department: A Case Report

**DOI:** 10.5811/cpcem.2020.7.47237

**Published:** 2020-07-30

**Authors:** Samuel Nesemann, Kimberly A. Hubbard, Mehdi I. Siddiqui, William G. Fernandez

**Affiliations:** * University of Texas Health San Antonio, Department of Emergency Medicine, San Antonio, Texas; † University of Texas Health San Antonio Long School of Medicine, Department of Emergency Medicine, San Antonio, Texas

**Keywords:** Rectal foreign body, emergency department

## Abstract

**Introduction:**

Rectal foreign bodies (RFB) pose a challenge to emergency physicians. Patients are not often forthcoming, which can lead to delays to intervention. Thus, RFBs require a heightened clinical suspicion. In the emergency department (ED), extraction may require creative methods to prevent need for surgical intervention.

**Case Report:**

The authors present a case of a successful extraction of a RFB in the ED and review of the literature.

**Conclusion:**

Retained RFBs are an unusually problematic reason for an ED visit. Thus, it is important for emergency physicians to be comfortable managing such cases appropriately.

## INTRODUCTION

Abdominal pain due to a retained rectal foreign body (RFB) is an unusually problematic complaint in the emergency department (ED) setting.^1^–^3^ The true incidence of retained RFB in the community is not currently known.^1^ A study done using two large hospitals in Southern California had an incidence of nearly one episode per month over nine years. This presentation is not a recent phenomenon; in fact, the earliest reports date back to the 16th century.^2^ Most RFBs are inserted for the purpose of autoerotic sexual gratification.^3^,^4^ Naturally, having a retained RFB often leads to some degree of embarrassment for the patient, which may result in reticence in providing a full account of the situation, which may impede the physician from obtaining an accurate history.^5^ Typically, patients with retained RFB present to the ED several hours after insertion following failed attempts at self-removal.^5^ In such cases, radiographic imaging is a key diagnostic modality. Successful management of RFB in the ED involves early diagnosis and triage for extraction. In this report, we describe an approach to extracting a retained RFB in the ED setting.

## CASE REPORT

A Hispanic female in her 40s presented to the ED approximately six hours after inserting a cylindrical deodorant container into her rectum. After discussing anal sex with friends, she became curious and inserted the deodorant canister into her rectum. The patient became distressed by her inability to remove the object and developed dull, diffuse lower abdominal pain that radiated to her rectum. Upon ED presentation, she was in moderate discomfort, lying in the lateral decubitus position.

On physical exam, the patient’s vital signs were normal; additionally, there was diffuse tenderness to palpation of the lower abdomen. On inspection of the perineal area, there were no signs of external trauma or other abnormalities noted. A hard, cylindrical structure was palpable approximately five centimeters (cm) into the rectum on digital rectal exam, posteriorly displaced from the anal orifice. The patient was given morphine four milligrams (mg) intravenously, and then an abdominal kidney-ureter-bladder (KUB) radiograph was ordered for evaluation of RFB. The key findings of the KUB included “a cylindrical lucency projecting over the rectum consistent with inserted foreign object” (Image).

Based on the patient’s presentation and radiographic findings, extraction of the canister in the ED was attempted. Prior to the procedure, the patient was placed on a cardiac monitor with pulse oximetry, and given supplemental oxygen before receiving lorazepam 2 mg for anxiety, and later 4 mg morphine sulfate for pain, to facilitate the procedure. To extract the canister, three successive methods were used. The patient was placed in a lateral decubitus position, and a lubricated finger was inserted into the rectum to locate the canister (manual extraction method). Once located, a second finger on the opposite hand was inserted into the anus to gain traction with one finger on each side of the canister and pull the object out of the anus. Unfortunately, the anal orifice did not allow enough space for two fingers to be inserted far enough to obtain traction sufficient for removal.

Next, a lubricated finger was used to guide a coudé catheter past the canister, using the first finger for guidance (coudé catheter method). Then, the catheter balloon was inflated with saline and traction applied to dislodge the canister. This was unsuccessful, likely because the catheter was not rigid enough to apply the necessary pressure for extraction. Finally, a lubricated finger was used to guide a set of ring forceps around the canister. Traction was applied to the forceps while squeezing to maintain contact with the canister (forceps method). This was attempted three times, but the forceps dislodged each time. On a fourth attempt, we maneuvered the canister from its posteriorly displaced position to a position in line with the anal orifice using the forceps. Once in this position, we applied gentle traction to remove the RFB; the deodorant canister measured approximately 11.5 cm in length by 3.5 cm in diameter.

**CPC-EM Capsule**

What do we already know about this clinical entity?

Despite careful emergemcy department (ED) management, certain factors reduce the odds of rectal foreign body (RFB) extraction. In such cases, endoscopic or surgical removal is necessary.

What makes this presentation of disease reportable?

This case highlights the challenge of RFB removal in the ED. In some cases, several attempts may be required to avoid the need for endoscopic or surgical extraction.

What is the major learning point?

In addition to detailed history-taking and adequate patient preparation, making time to allow for an unhurried extraction is critical to success in RFB cases.

How might this improve emergency medicine practice?

We highlight some best practices as well as key challenges to the safe removal of RFB in the ED. Also, we list conditions where additional computed tomography imaging is advised.

Following the extraction, the patient had complained of persistent abdominal discomfort. Therefore, an intravenous contrast-enhanced computed tomography (CT) of the abdomen and pelvis was ordered to evaluate for perforation or damage to the bowel. Only mild rectal wall thickening without free air or signs of perforation was seen on CT.

## DISCUSSION

### Patient Evaluation

The first step in patient evaluation requires a focused history with an emphasis on the nature of the RFB and manner of insertion. Although not so in the present case, the majority of patients presenting with RFB are white males in their 40s.^6^,^14^–^15^ While many RFBs are smooth and egg-shaped, which facilitates insertion, some may have sharp edges or are easily fragmented.^6^ Thus, some recommend abdominal radiographic imaging prior to digital rectal exam (DRE) in order to identify sharp edges on the RFB that could result in provider injury.^6^ Additionally, imaging may identify free air, and help to assess the size and depth of the RFB.^6^ RFBs that contain sharp edges, are over 10 cm, have entered the sigmoid colon, or that have been retained for two or more days are less likely to be extracted in the ED, and may require endoscopic or surgical removal.^6^–^7^, ^14^–^15^ Importantly, “body-packers” – those who conceal illicit drugs by swallowing latex balloons filled with such illegal substances in smuggling attempts – will require close monitoring in the event that such balloons break during transit through the bowels, as extraction should not be attempted in these patients.^1^ Additionally, the risk of perforation is not limited to sharp or easily fragmented RFBs; it also is related to the force of insertion.^8^ Obtaining detailed information about the size and shape of the RFB, as well as the manner and circumstances with which it was introduced is imperative, as most failures of manual extraction in the ED can be predicted preoperatively.^9^

Next, imaging should be obtained. It is important to first assess for perforation both clinically and via imaging, such as an upright chest radiograph. RFB perforation is a potential surgical emergency and should result in immediate surgical intervention.^2^, ^6^–^8^,^13^–^15^ In addition to assessing perforation, imaging can also determine the general location of the RFB within the abdomen, which affects disposition. For instance, if the RFB is proximal to the rectosigmoid junction, endoscopic removal is recommended.^1^ However, if it is distal to this point, a DRE should be performed. If the RFB cannot be palpated on DRE, manual extraction should not be attempted, and a surgeon should instead be consulted for either endoscopic or operative removal.^2^,^7^,^10^, ^14^–^15^

### Techniques

There are several key principles of managing RFBs within the ED to optimize successful extraction. These include minimizing cross-sectional area, employing visualization during extraction, overcoming suction, and limiting procedure time.^8^ First, it is important to grasp the RFB securely. Broadly, the literature describes the use of forceps, Foley catheters, and bimanual manipulation for extraction.^11^ Several reports mention the use of obstetric forceps as grasping tools, 1,6–9,11 while others suggest the use of endoscopic snares to grasp the object.^1^–^4^,^6^ While there is little consensus in the literature regarding specific techniques within each category of grasping tools, the vast majority of reports suggest first attempting bimanual manipulation, and then proceeding to the use of forceps before involving endoscopy. If the object is difficult to remove with simple grasping, it is likely that the suction effect must be overcome. This is accomplished in several different ways, including the use of a Foley catheter, endotracheal tube, or air insufflation during endoscopy.^7^,^12^

Additionally, to increase the success of RFB removal during an extraction attempt, it is important to keep the patient calm, and to control their pain. If they can tolerate the procedure without being sedated, they can actively aid in removal by performing the Valsalva maneuver at a specific time.^7^ However, given the discomfort in the removal process, sedative agents are often necessary, and may include procedural sedation or perianal local anesthesia,^5^–^6^ although this may be beyond the scope of ED management.^7^ Additionally, the generous use of lubricant^1^ and placing the patient in the lithotomy position may also be used to facilitate extraction.^5^ Regardless of the outcome of the RFB extraction attempt, the patient should be observed for several hours with repeated abdominal exams for signs of peritonitis from perforation.^6^,^14^–^15^ Any evolving changes in the abdominal exam or other concerning findings (e.g., vital sign changes, vomiting) should warrant abdominal CT imaging and urgent surgical consultation.^6^, ^14^–^15^ Finally, after discharge, patients should have close follow-up for any subsequent post-extraction complications.

## CONCLUSION

Retained RFBs are an unusual reason for ED presentation. However, it is important for emergency physicians to be comfortable managing these patients appropriately. Most cases can be successfully managed in the ED via forceps-assisted manual extraction, effectively removing the object with minimal long-term complication. Some cases will require referral for endoscopic or operative extraction.

## Figures and Tables

**Image:**
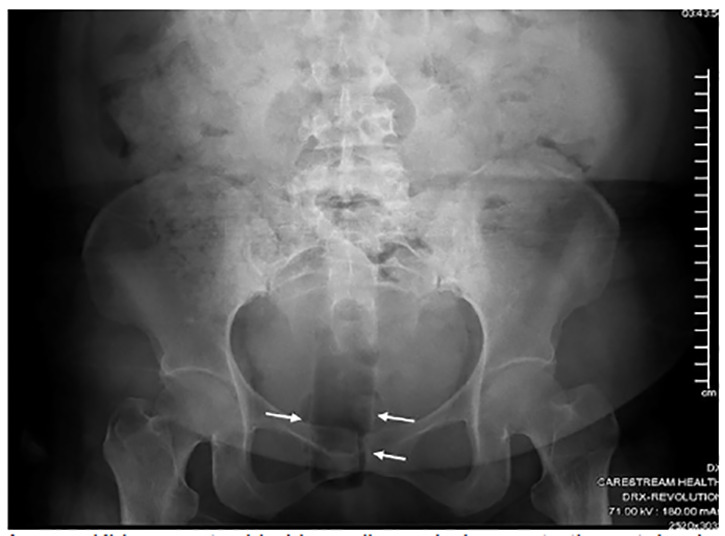
Kidney-ureter-bladder radiograph demonstrating retained rectal foreign body as lucency in rectum (arrows).
